# Association of Primary Hypertension and Risk of Cerebrovascular Diseases with Overweight and Physical Activity in Korean Women: A Longitudinal Study

**DOI:** 10.3390/healthcare9091093

**Published:** 2021-08-24

**Authors:** Nan Su, Yonghwan Kim, Youngin Won

**Affiliations:** 1 School of Physical Education (Main Campus), Zhengzhou University, Zhengzhou 450001, China; Sunan0702@whu.edu.cn; 2 Department of Physical Education, Gangneung-Wonju National University, Gangneung 25457, Korea; yhkim@gwnu.ac.kr; 3 Department of Rehabilitation Personal Training, Konyang University, Nonsan 32992, Korea

**Keywords:** primary hypertension, cerebrovascular disease, physical activity, obesity, relative risk

## Abstract

Cerebrovascular diseases include stroke, intracranial stenosis, aneurysms, and vascular malformations; primary hypertension is typically associated with cerebrovascular disease. The incidence of these diseases is higher in men than in women, and low physical activity and obesity are known to increase the risk of cerebrovascular disease. This study aimed to longitudinally analyze the adjusted relative risk (ARR) of primary hypertension and cerebrovascular diseases, in relation to body mass index (BMI) and physical activity (PA), in Korean women. The study retrieved the data of 1,464,377 adult Korean women (aged 50–79 years), who participated in the national health screening program from 2002 to 2003. The participants had no history of primary hypertension or cerebrovascular diseases, and were followed up by the International Statistical Classification of Diseases and Related Health Problems (ICD) until 2013. The participants were divided into the following groups: normal weight (18.5–24.9), overweight (25.0–29.9), and obese (≥30.0) kg/m^2^, based on the World Health Organization (WHO) classification. The frequency of PA (days) was determined using a physical activity questionnaire, and defined as low (0–2), medium (3–4), and high (5–7) days. The RR was calculated using Cox regression. Three models were created based on the adjusted variables. The ARR for hypertension was 0.933 (95% CI; 0.920–0.955, *p* < 0.001) in obese patients with medium PA. Primary hypertension was lower (ARR: 0.943; 95% CI; 0.928–0.961, *p* < 0.001) in overweight participants with medium PA, than in those with low PA. The incidence of cerebrovascular disease was lower in overweight individuals with medium PA (ARR: 0.945, 95% CI; 0.925–0.976, *p* < 0.001), than in those with low PA. The risk of cerebrovascular disease was reduced in normal-weight participants with medium PA (ARR: 0.889; 95% CI: 0.854–0.919; *p* < 0.001), than in those with high PA (ARR 0.913; 95% CI; 0.889–0.953, *p* < 0.001). In the obese group, there was no significant difference in the risk of cerebrovascular disease, based on the frequency of PA. In conclusion, the relative risk of primary hypertension in women was lower with moderate activity than with low activity, in the normal-weight and overweight groups. The relative risk of cerebrovascular disease was lower in the participants with moderate and high activity than in those with low activity, even at normal weight. In obese individuals, moderate and high activity reduced cerebrovascular disease compared to low activity. Therefore, regardless of obesity, PA may contribute to the prevention of primary hypertension and cerebrovascular disease in adult women.

## 1. Introduction

Cerebrovascular diseases include subarachnoid hemorrhage, intracerebral hemorrhage, and cerebral infarction, commonly called stroke [1]. Cerebrovascular diseases are the second leading cause of death globally, and primary hypertension is the predominant risk factor for cerebrovascular diseases [2,3,4]. In 2015, the death rates from stroke were 73.3 and 29.6 per 100,000 persons in the United States and Korea, respectively [5,6]. Cerebrovascular disease is a critical condition in healthcare because of the tremendous economic burden and physical impact; stroke, in particular, decreases the quality of life of individuals suffering from sequelae, such as hemiplegia [4,7].

Some risk factors for cerebrovascular disease and primary hypertension are non-modifiable, such as age, sex, and genetics [8]. Meanwhile, the modifiable risk factors include being overweight, having low physical activity, smoking, and having a high alcohol consumption [9]. Specifically, physical activity not only lowers the rate of hypertension, but also reduces the rates of cardiovascular disease, by 4–5%, and stroke, by 6–8%, in both hypertensive and normotensive individuals [10]. Alcohol consumption increased the risk of stroke by 3.62 times when drinking 250 g per week, compared to less than 100 g [11]. Moreover, the prevalence of stroke decreased by 51% through physical exercise, which increased 2.01 times for overweight individuals and 1.84 times for smokers [9].

In generally, it has been reported that stroke mortality in men is 1.5 to 2 times higher than in women [12]. However, in the Korean study, the mortality rate per 100,000 persons was 72.9 for men and 82.1 for women in 2002, and it was 51.5 for men and 54.8 for women in 2010, which was higher in women than men [13].

Despite the lower alcohol intake and smoking rate among Korean women than men [14]. The prevalence of obesity among Korean women is lower than that of men, until the age of 50, but from the age of 60, women appear to be higher than men [15]. Furthermore, women’s PA participant rate is generally low in PA. In Korea, the rate of involvement in aerobic exercise in 2014 was 62.0% in men and 54.7% in women, and these rates declined to 52.5% and 46.4%, respectively, in 2016 [16]. In a study conducted in the United States, 60% of respondents said that they did not exercise regularly, and 25% reported that they did not exercise at all [16,17,18]. Notably, low participation in PA is more prevalent in women than in men. According to a previous study, men participate more in leisure activities than women, and this difference increases with age [19]. Moreover, women have a lower socioeconomic status and access to medical care than men, and these social factors are the reasons for the low health status of women [20].

Therefore, this study investigated the relative risk of primary hypertension and cerebrovascular disease in Korean women, using obesity and physical activity variables. Most of the previous research involved sampling studies, which are useful for characterizing specific groups, but there are limitations in identifying the public health of an entire country. Therefore, this study was conducted using a national health screening census database, involving all citizens. The study aimed to evaluate the relative risks of primary hypertension and cerebrovascular diseases, according to the obese class and PA, in adult women.

## 2. Methods

### 2.1. Participants and Data Sources

**Figure 1:**
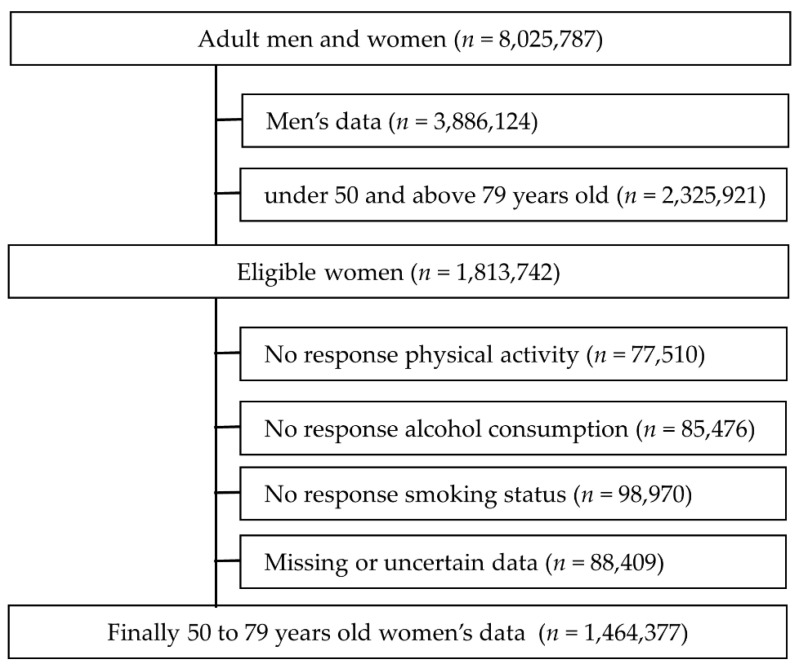
Participant’s exclusion and inclusion process.

Data from the national health screening and the Korean National Health Insurance Service (KNHIS) databases, which contain information regarding diseases of patients, were merged. This study initially collected data on 8,025,787 adults aged 20–79 years who participated in national health screening between 2002 and 2003, with no history of primary hypertension or cerebrovascular disease. Men (*n* = 3,886,124) and women under 50 and over 79 years old (*n* = 2,325,921) were excluded. Participants who did not complete the questionnaire were also excluded. Physical activity (*n* = 77,510), alcohol consumption (*n* = 85,476), smoking (*n* = 98,970). Last, missing data or uncertain information were also deleted (*n* = 88,409). Finally, the data used for the analysis were 1,464,377 women aged 50 to 79 years (Figure 1). Data analysis was provided by the KNHIS. This study was approved by the Institutional Review Board of Yonsei University Gangnam Severance Hospital (3-2015-0059). The research was funded by the KNHIS research fund and service support in 2015 [21].

### 2.2. Diagnosis and Follow-Up

The disease code followed the classification criteria of ICD and was defined as I10–I13 for primary hypertension and I60–I69 for cerebrovascular disease [22]. Korea has a national health insurance system, and every patient who visits a hospital is assigned a diagnosis code and dates are stored in the KNHIS database. Therefore, in this study, the KNHIS database was used to follow-up patients until the day of occurrence of the disease code.

The criteria for the occurrence of hypertension are SBP ≥ 140 mmHg and DBP ≥ 90 mmHg according to the guidelines of the Seventh Joint National Committee on Prevention, Detection, Evaluation, and Treatment of High Blood Pressure (JNC 7) [23]. Cerebrovascular disease codes are assigned when occlusion, stenosis, aneurysm, and hemorrhage are detected through imaging and medical examination by a specialist [24]. 

### 2.3. Health Screening

Every two years, free health screening is offered to adults with Korean citizenship and is conducted at local hospitals designated by the KNHIS. For accurate testing, participants must fast after 9 p.m. the previous day, for at least 8 h until morning. The test is performed with the participant wearing a light gown provided by the health screening center. The procedure confirms identification, completes a health and medical history questionnaire, and conducts a physician consultation. General medical test includes the measurement of height, weight, blood pressure, electrocardiogram, X-ray, oral examination, hearing, vision, blood and urine analysis, gastroscopy and physician consultation. After all examinations are completed, the doctor confirms safety, and then leaves the hospital.

### 2.4. BMI: Normal, Overweight and Obese

Height and weight measuring equipment are certified by the government’s scientific verification agency. BMI was calculated as the weight (kg) divided by the square of height (m^2^). Classification based on the BMI was performed as described by the World Health Organization (WHO). Participants were divided into normal weight (18.5–24.9 kg/m^2^), overweight (25.0–29.9 kg/m^2^), and obese (≥30.0 kg/m^2^) groups [25]. Individuals with BMI < 18.5 kg/m^2^ were excluded. 

### 2.5. Physical Activity, Smoking Status, and Alcohol Consumption Risk, Family Monthly Income

Participants used a self-filling method with a paper questionnaire and a pencil. Exceptionally, staff helped when self-filling was difficult due to lack of understanding, low vision, or other health reasons. Patients were also provided with a survey concerning physical activity, smoking, and drinking. The physical activity questionnaire was constructed based on the Paffenbarger physical activity questionnaire [26]. This questionnaire comprises eight questions, and the validity of the question regarding the frequency of exercise-induced sweating has been verified [27]. We translated and applied the questionnaire as relevant to this study. The American College of Sports Medicine (ACSM) recommends moderate-intensity exercise for at least 5 days per week and high-intensity exercise for at least 3 days per week [28]. Exercise intensity was not investigated in this study; therefore, the final classification was defined as low PA (0–2), medium PA (3–4) and high PA (5–7) based on the number of days of involvement in PA.

The smoking and drinking survey was completed according to the WHO guidelines [29,30]. For smoking-related questions, current smoking status was queried, amount and duration of smoking were investigated for current smokers, and time of quitting was investigated for past smokers. In this study, never smoked, quit smoking, and current smoking status were used.

Alcohol consumption and risk were also classified based on the WHO guidelines [30]. In the questionnaire, the type, frequency of drinking per week, and amount of alcohol consumed were evaluated. The amount of pure alcohol was calculated according to the type of alcohol, with 1–20 g, 21–40 g, and 41–60 g classified as low, medium, and high risk, respectively.

Family monthly income was entered directly, and the total amount of expenses that family members earn for an average of one month was entered.

### 2.6. Data Analysis

SAS (version 9.4; SAS Institute Inc., Cary, NC, USA) was used for the analysis. Normal distribution was confirmed using the Kolmogorov–Smirnov test (*p* > 0.05), and continuous variables including age, height, and weight were reported as means and standard deviations; the one-way ANOVA and Bonferroni post hoc analysis were performed to identify differences in general characteristics according to BMI classification. Categorical variables such as physical activity, smoking status, and alcohol consumption risk were analyzed using the chi-square test. Cox regression analysis was performed to derive the relative risk, which is the main analytical method used in this study. We evaluated the relative risk (RR) of the following three adjusted models: model 1: age; model 2: age, smoking, alcohol, family monthly income; model 3: age, smoking, alcohol consumption, family monthly income, total cholesterol, fasting glucose, AST, and ALT. The reference group for PA was designated as the low-activity group, and the relative risk was calculated compared to medium- and high-activity levels. Significance was set at *p* < 0.05 and the confidence interval (CI) was set to 95% and presented as lower and upper values. 

## 3. Results

### 3.1. General Characteristics of Participants

The BMI was normal in 57.2% of the participants, whereas 39.3% were overweight, and 3.5% were obese (Table 1). The participants were older in the overweight and obese groups (66.4 ± 6.5 and 67.1 ± 7.1 years, respectively) compared to those in the normal-weight group (59.2 ± 7.3 years). The total cholesterol levels were 192.7 ± 41.3, 217.3 ± 59.6, and 219.1 ± 60.1 mg/dL, in the normal, overweight, and obese groups, respectively (*p* < 0.001). The systolic blood pressure (SBP) and diastolic blood pressure levels for the normal, overweight, and obese groups were (115.3 ± 11.3 vs. 119.3 ± 11.8 vs. 123.4 ± 12.5 mmHg), and (75.7 ± 6.1 vs. 77.1 ± 7.9 vs. 81.6 ± 8.3 mmHg), respectively. Aspartate transaminase (AST) (22.9 ± 18.3 vs. 26.7 ± 21.7 vs. 29.3 ± 27.7 U/L), alanine transaminase (ALT) (20.1 ± 21.3 vs. 24.7 ± 22.6 vs. 30.4 ± 26.1 U/L), and fasting glucose (92.2 ± 23.5 vs. 99.7 ± 32.7 vs. 109.5 ± 37.1 mg/dL) showed significant differences among the normal, overweight, and obese groups, respectively (*p* < 0.001).

Physical activity more than three days per week was reported by 13.8% of the participants for the normal-weight group, 16.6% for the overweight group, and 14.4% for the obesity group (Table 2). The current smoking rate of the obesity group (3.9%) was higher than that of the normal-weight group (3.7%). Overall, however, women had a very high rate (94.3–95.6%) of no experience smoking. More than 90% of the subjects did not drink alcohol at all, regardless of their BMI. Only 1.1% of the normal-weight group, 1.6% of the overweight group, and 1.9% of the obesity group fell into the drinking high-risk group (*p* < 0.001). The family monthly income was also higher in the normal group, and significantly lower in the obese group (*p* < 0.001).

### 3.2. Incidence of Cerebrovascular Diseases and Adjusted Relative Risk According to BMI and Physical Activity Classifications

The incidence of cerebrovascular disease was 5.7% for low, 5.4% for medium, and 7.6% for high PA in the normal-weight participants (Table 3), whereas in the overweight individuals, the incidence was 9.4%, 8.6%, and 10.9% in the low, medium, and high PA groups, respectively. In model 3, the adjusted relative risk (ARR) primarily decreased. Except for the obese group, the ARR decreased in the normal and overweight groups; the ARR decreased to 0.889 in the medium PA group and 0.913 in the high PA group, compared to the low PA group. The ARR in the overweight participants with medium PA decreased to 0.945 compared to those with low PA.

### 3.3. Incidence of Primary Hypertension and Adjusted Relative Risk According to BMI and Physical Activity Classifications

The normal-weight group exhibited the following primary hypertension rates, based on physical activity: low PA, 17.6%; medium PA, 17.9%; and high PA, 24.6% (Table 4). In the overweight group, the rates of hypertension were 40.1, 36.5, and 44.7%, respectively, and for the obese group, the rates were 48.7, 44.9, and 55.8%, respectively. In the overweight group, the ARR for the medium PA group decreased to 0.943 (95% CI; 0.928–0.961, *p* < 0.001), compared to the low PA group. The ARRs for the obese participants with medium and high PA were 0.933 (95% CI; 0.920–0.955, *p* < 0.001) and 0.981 (95% CI; 0.971–0.997, *p* < 0.001), respectively.

## 4. Discussion

Cerebrovascular diseases are serious medical conditions with a high mortality rate, and hypertension and diabetes are considered significant risk factors. The incidence of cerebrovascular disease increases with age, and men are more affected than women [31]. Further, the mortality rate from cerebrovascular disease is the third highest in both men and women in Korea, the fourth and fifth in women and men in the United States, and the second in the world [4,32,33]. Hypertension is a strong risk factor for cerebrovascular diseases. In a previous study, the incidence of cerebrovascular disease in individuals with high blood pressure was 2.68 times higher than that in healthy persons [9]. 

Therefore, the management of risk factors is required to prevent cerebrovascular disease and hypertension. The representative risk factors include low PA and obesity, and it is necessary to engage in PA and maintain normal weight, along with a balanced diet [8,9]. A meta-analysis reported that the prevalence of stroke decreased by 51% through physical exercise, which increased 2.01 times for overweight individuals and 1.84 times for smokers [9]. Therefore, this study analyzed the relative risk for cerebrovascular disease and primary hypertension according to the obese level and PA.

One of our main findings was a decrease in primary hypertension and cerebrovascular disease ARR as physical activity increased. This result is similar to that reported in previous studies [34,35,36,37]. Most cerebrovascular disease studies reported positive results for PA. In a study by Barengo et al. [34], women who participated in high leisure-time PA had a stroke hazard ratio of 0.43, compared to those with low PA. In a 10-year follow-up study on women >50 years of age in Norway, it was reported that, compared to low PA, medium and high PA reduced stroke by 23% and 48%, respectively [35]. A Japanese study also classified PA into quartiles, and after a 12-year follow-up study, the highest activity group was reported to observe a reduced risk of stroke by 13%, compared to that in the lowest-activity group [36]. A Taiwan study has reported that the benefit of PA for cerebrovascular disease is not only a preventive function, but can also reduce hemorrhagic infarct by 29% and mortality by 8–17% after stroke [37]. A study that was conducted in the United States also found that the inactive group had a 1.2-fold increased risk of stroke compared to the group that was physically active four or more times per week [38]. 

In a primary hypertension RR study, it was found that the number of persons participating in walking/running and sports had decreased risk by 13% and 24%, respectively, which was lower than sedentary individuals [39]. A meta-analysis of 22 studies also reported that PA reduced hypertension by 6% [40], while another meta-analysis on PA and hypertension showed that the RR for those with high PA ranged from 0.64 to 0.92, and that for those with moderate PA decreased to 0.65 [41]. Camoes et al. [42] demonstrated that the moderate-activity group’s RR was 0.74, and the RR of the high-activity group was approximately 0.77. Diaz et al. [43] found that the intermediate and ideal groups of moderate–vigorous PA had a lower hypertension hazard ratio, by 0.84 and 0.76, than the low PA group, respectively. Even in the presence of hypertension, the mortality rate produced a positive result, due to PA. A study that was conducted in the city of Copenhagen, analyzed death from hypertension, with regard to PA. As a result, death in the moderate- or high-activity group decreased by 31% and 28% in the first and second stages of hypertension, respectively, compared to that in the inactive group [44].

However, there were some unexpected results in this study that differed from those traditionally known. First, for some results, the PA and ARR relationships were not significant. There was no significant difference between the normal and overweight patients with high PA, in terms of hypertension. Moreover, no significant ARR, related to cerebrovascular disease, was noted, according to PA in the obese group. Although, these results are not common, and they have rarely been reported in previous studies. A study by Lu et al. [45] found that there was no significant relationship between the PA level and the hazard ratio of hypertension when the mean 4.5 years was followed. A study of 50–79-year-old Hispanic postmenopausal women did not show significant results in hypertension prevalence, following a week of PA [46]. Moreover, some studies related to cerebrovascular diseases have not indicated the significance of PA. A study spanning 15.5 years showed that women with light PA had a 1.95-fold increase in stroke compared to those with moderate PA, but men did not show any significant results [47]. In another study, which followed up women for an average of 11.9 years, the age-adjusted RR in women with high activity was decreased to 0.65 compared to those with low activity. However, after adjustment for stroke-related factors, such as BMI, nutrition, cholesterol, alcohol, and smoking, no significant results were observed according to PA [48]. 

These results indicate that the risk factors for cerebrovascular disease or primary hypertension are very diverse. These results indicate that, in addition to PA, a wide variety of risk factors further affect hypertension and cerebrovascular disease, and there are limits to controlling and analyzing these factors. JNC 7 contains a similar statement. The report stated that PA can lower blood pressure by 4–9 mmHg, dietary approaches to stop hypertension (DASH) by 8–14 mmHg, and weight loss by 5–20 mmHg [49].

The overweight group displayed higher PA in this study, and this result was unexpected. In our study, 13.8%, 16.6%, and 14.4% normal, overweight, and obese participants, respectively, undertook medium and high PA. This result is perhaps attributed to obese participants trying to lose weight. A study in the United Kingdom reported that the overweight group had a slightly higher rate of meeting aerobic guidelines, at 72%, than the normal group, at 70% [50]. Further, there was no significant difference according to BMI, regarding fulfillment of the WHO recommendation of 150 min of moderate exercise or 75 min of high-intensity exercise [51,52]. As a limitation of this study, the group that performed mostly light walking and low-intensity exercise was not considered, because our study surveyed the level of sweating.

Despite some unexpected results, this study reports positive effects of PA on primary hypertension, reduction in cerebrovascular disease, and has several strengths. This was a large-scale longitudinal study on the RR of primary hypertension and cerebrovascular disease in relation to obesity and PA in women. In particular, considering the population of Korea, about 1.8 million people comprises a very large cohort. Moreover, one of the characteristics of this study was that the BMI classification was first conducted without statistically including BMI as a covariate. We also analyzed the low incidence of cerebrovascular disease and primary hypertension in women. Although the incidence in women is low, the severity is not low. According to the Korean national health and nutrition examination survey, the prevalence of hypertension was 35.0% for men and 22.9% for women in 2016 [16], but the hypertension mortality rate was 7.4 in men and 14.8 in women per 100,000 persons in 2007, but decreased to 6.9 in men and increased to 15.6 in women in 2017 [32]. Although the smoking and drinking rates are lower in women than in men, the reason for a high mortality rate may be due to less PA. The low rate of participation of women in PA has been reported not only in Korea, but globally [16,19,53]. 

ACSM’s guidelines recommend PA to prevent cardiovascular disease or related factors; individuals should perform 150–300 min of moderate exercise or 75–150 min of vigorous exercise per week, and they should exercise three or more days per week [28]. As an effect of the cardiovascular disease education program, as a result of diet and nutrition education for postmenopausal women for 2 years, it was possible to induce significantly lower calorie intake in the intervention group than in the control group [54], and the 3-year cardiovascular disease management program led to improvements in the negative factors for cardiovascular disease in black women [55].

Although this study applied longitudinal data analysis using big data, there were several limitations. BMI was used as a variable and was based only on initial measurements. Therefore, if a participant subsequently lost weight, they should be placed in the normal-weight category; however, the change was not reflected in their placement in the study. The average BMI of the overweight group was 27.4; thus, it is possible that some of the participants may have lost weight, such that they returned to their normal BMI range. Moreover, the database of health screening was collected medical results from local hospitals across the country. Therefore, it is a limitation of this study that diagnostic medical devices, including height and weight measuring machines, were not unified. Of course, diagnostic devices have been certified by government agencies, but errors may exist, depending on the device. In this study, a questionnaire on exercise was conducted using a self-report method, to determine the number of days per week of sweat-inducing exercise. Various forms of physical activity, such as sports, recreational activities, and aerobic and strength training, were not investigated. Moreover, information on sedentary life and occupational activities is not available. Information on psychological status, such as mental or quality of life, could not be confirmed. Although there are many factors influencing cerebrovascular disease and hypertension, this study focused too much on physical activity and BMI, so the RR analysis for alcohol and smoking was not analyzed. Another limitation of this study is that obesity, cerebrovascular disease, and primary hypertension are all affected by dietary habits. However, the data in this study did not allow for diet to be factored in, because there are no data on calories and dietary patterns. Subsequent studies should include diverse variables, such as diet, occupational activity, smoking, and drinking. It is also necessary to analyze characteristics not only by sex, but also by age group. Additionally, it would be very meaningful to analyze the mortality rate from cerebrovascular disease, according to physical activity and BMI.

## 5. Conclusions

The obese group was older, with higher blood pressure, total cholesterol, and fasting blood glucose than the normal and overweight groups. The adjusted relative risk of primary hypertension decreased with medium physical activity in the normal and overweight groups, compared to that in the low-activity group. Moreover, the risk of cerebrovascular disease was also reduced in the medium- and high-activity groups compared to that in the normal and overweight groups. These results indicate that physical activity can prevent primary hypertension and cerebrovascular disease.

## Figures and Tables

**Table 1:** Characteristics of participants based on BMI classification.

	Normal	Overweight	Obese	*p*-Value
*n*, (%)	837,623 (57.2%)	575,500 (39.3%)	51,253 (3.5%)	
Age, years	59.2 ± 7.3	66.4 ± 6.5 ^a^	67.1 ± 7.1 ^b,c^	<0.001 *
Height, cm	157.9 ± 6.3	155.3 ± 5.6 ^a^	154.1 ± 5.1 ^b^	<0.001 *
Weight, kg	55.2 ± 6.2	66.1 ± 6.8 ^a^	79.9 ± 8.3 ^b,c^	<0.001 *
BMI, kg/m^2^	22.1 ± 2.4	27.4 ± 1.7 ^a^	33.6 ± 2.3 ^b,c^	<0.001 *
SBP, mmHg	115.3 ± 11.3	119.3 ± 11.8 ^a^	123.4 ± 12.5 ^b,c^	<0.001 *
DBP, mmHg	75.7 ± 6.1	77.1 ± 7.9 ^a^	81.6 ± 8.3 ^b,c^	<0.001 *
Total cholesterol, mg/dL	192.7 ± 41.3	217.3 ± 59.6 ^a^	219.1 ± 60.1 ^b,c^	<0.001 *
Fasting glucose, mg/dL	92.2 ± 23.5	99.7 ± 32.7 ^a^	109.5 ± 37.1 ^b,c^	<0.001 *
AST, U/L	22.9 ± 18.3	26.7 ± 21.7 ^a^	29.3 ± 27.7 ^b,c^	<0.001 *
ALT, U/L	20.1 ± 21.3	24.7 ± 22.6 ^a^	30.4 ± 26.1 ^b,c^	<0.001 *

* *p* < 0.05; ^a^ normal vs. overweight; ^b^ normal vs. obese; ^c^ overweight vs. obese. BMI, body mass index; SBP, systolic blood pressure; DBP, diastolic blood pressure; HbA1c, hemoglobin A1c; AST, aspartate transaminase; ALT, alanine transaminase.

**Table 2:** Physical activity, smoking status, alcohol consumption risk, and family monthly income based on BMI classification.

	Normal	Overweight	Obese	*p*-Value	*p*-Value
Physical Activity, (%)					
Low	86.2	83.4	85.6	normal vs. overweight, *p* < 0.001 * normal vs. obese, *p* < 0.001 * overweight vs. obese, *p* = 0.003	<0.001 *
Medium	7.3	8.0	6.5		
High	6.5	8.6	7.9		
Smoking status, (%)					
Never	94.3	95.6	94.5	normal vs. overweight, *p* < 0.001 * normal vs. obese, *p* < 0.001 * overweight vs. obese, *p* < 0.001 *	<0.001 *
Quit smoking	2.0	1.4	1.6		
Current smoker	3.7	3.0	3.9		
Alcohol consumption risk, (%)					
None	90.9	91.0	91.3	normal vs. overweight, *p* < 0.001 * normal vs. obese, *p* < 0.001 * overweight vs. obese, *p* = 0.007	<0.001 *
Low risk	7.4	6.6	5.9		
medium risk	1.1	1.6	1.9		
High risk	0.6	0.8	0.9		
Family monthly income, KW	2,799,315	2,710,819 ^a^	2,594,100 ^b,c^	-	<0.001 *

* *p* < 0.05; physical activity (PA) was defined as low: 0–2 days, medium: 3–4 days, and high: 5–7 days; alcohol consumption was classified according to the WHO; KW: Korean currency (Won). ^a^ normal vs. overweight; ^b^ normal vs. obese; ^c^ overweight vs. obese.

**Table 3:** Incidence of cerebrovascular diseases and adjusted relative risk based on BMI and physical activity classifications.

	P.A	Incidence (%)	Model 1		Model 2		Model 3	
			ARR (95% CI)	*p*	ARR (95% CI)	*p*	ARR (95% CI)	*p*
Normal BMI 18.5–24.9	Low	5.7	1		1		1	
	Medium	5.4	0.931 (0.913–0.959)	<0.001 *	0.896 (0.860–0.933)	<0.001 *	0.889 (0.854–0.919)	<0.001 *
	High	7.6	0.956 (0.922–0.986)	<0.001 *	0.956 (0.921–0.969)	<0.001 *	0.913 (0.889–0.953)	<0.001 *
Overweight BMI 25.0–29.9	Low	9.4	1		1		1	
	Medium	8.6	0.839 (0.806–0.869)	<0.001 *	0.959 (0.931–0.985)	<0.001 *	0.945 (0.925–0.976)	<0.001 *
	High	10.9	1.080 (0.894–1.230)	0.090	0.978 (0.964–1.009)	0.212	0.980 (0.964–0.991)	<0.001 *
Obese BMI ≥ 30.0	Low	9.3	1		1		1	
	Medium	7.5	1.112 (0.810–1.094)	0.893	1.001 (0.953–1.059)	0.981	0.997 (0.943–1.129)	0.846
	High	10.8	1.125 (0.911–1.235)	0.104	1.041 (0.995–1.086)	0.112	1.011 (0.976–1.063)	0.095

* *p* < 0.05. Physical activity (PA) was defined as low (0–2 days), medium (3–4 days), and high (5–7 days) adjusted for age, smoking, alcohol consumption, and family income. ARR: adjusted RR. Model 1: adjusted for age. Model 2: adjusted for age, smoking, alcohol, family monthly income. Model 3: adjusted for age, smoking, alcohol consumption, family monthly income, total cholesterol, fasting glucose, AST, ALT.

**Table 4:** Incidence of primary hypertension and adjusted relative risk according to BMI and physical activity classifications.

	PA	Incidence (%)	Model 1		Model 2		Model 3	
			ARR (95% CI)	*p*	ARR (95% CI)	*p*	ARR (95% CI)	*p*
Normal BMI 18.5–24.9	Low	17.6	1		1		1	
	Medium	17.9	0.995 (0.964–1.021)	0.125	0.986 (0.961–1.003)	0.159	0.985 (0.963–0.997)	<0.001 *
	High	24.6	1.085 (0.992–1.287)	0.098	0.993 (0.972–1.016)	0.451	0.991 (0.967–1.029)	0.234
Overweight BMI 25.0–29.9	Low	40.1	1		1		1	
	Medium	36.5	0.900 (0.847–0.931)	<0.001 *	0.946 (0.929–0.977)	<0.001 *	0.943 (0.928–0.961)	<0.001 *
	High	44.7	0.993 (0.942–1.059)	0.094	1.001 (0.988–1.030)	0.270	0.999 (0.988–1.019)	0.129
Obese BMI ≥ 30.0	Low	48.7	1		1		1	
	Medium	44.9	0.902 (0.821–0.969)	<0.001 *	0.941 (0.921–0.966)	<0.001 *	0.933 (0.920–0.955)	<0.001 *
	High	55.8	1.053 (0.927–1.199)	0.198	0.985 (0.974–0.995)	0.002 *	0.981 (0.971–0.997)	<0.001 *

* *p* < 0.05. Physical activity (PA) was defined as low (0–2 days), medium (3–4 days), and high (5–7 days). ARR: adjusted RR. Model 1: adjusted for age. Model 2: adjusted for age, smoking, alcohol, family monthly income. Model 3: adjusted for age, smoking, alcohol consumption, family monthly income, total cholesterol, fasting glucose, AST, ALT.

## Data Availability

The data are not publicly available due to privacy or ethical reasons.
